# Bridging evidence and practice: implementation science and bundled care strategies for fluid management in critically ill neonates

**DOI:** 10.3389/fmed.2026.1706165

**Published:** 2026-02-25

**Authors:** Amelia C. Pak, Javier A. Neyra, Jeremiah R. Brown, Colm P. Travers, Michelle C. Starr, Matthew W. Harer, Iben Sullivan, Todd A. MacKenzie, Tiago K. Colicchio, Russell Griffin, David J. Askenazi

**Affiliations:** 1 Department of Pediatrics, Division of Pediatric Nephrology, University of Alabama at Birmingham, Birmingham, Alabama, AL, United States; 2 Department of Nephrology, University of Alabama at Birmingham, Birmingham, AL, United States; 3 Department of Epidemiology, Dartmouth Center for Implementation Science, Dartmouth College Geisel School of Medicine, Hanover, NH, United States; 4 Department of Pediatrics, Division of Neonatology, University of Alabama at Birmingham, Birmingham, AL, United States; 5 Department of Pediatrics, Division of Pediatric Nephrology, Indiana University School of Medicine, Indiana, IN, United States; 6 Department of Pediatrics, Division of Neonatology, University of Wisconsin School of Medicine and Public Health, Madison, WI, United States; 7 Department of Biomedical Data Science, Dartmouth College Geisel School of Medicine, Hanover, NH, United States; 8 Department of Biomedical Informatics and Data Science, University of Alabama at Birmingham, Birmingham, AL, United States; 9 Department of Pediatrics, Department of Epidemiology, University of Alabama at Birmingham, Birmingham, AL, United States

**Keywords:** bundled care interventions, critically ill neonates, dissemination and implementation research, evidence-based practice, fluid management protocol, fluid overload, implementation science, pragmatic clinical studies

## Abstract

Fluid overload (FO) is a common and modifiable risk factor in critically ill neonates. FO is associated with prolonged mechanical ventilation, multi-organ dysfunction, and increased mortality. Despite substantial observational evidence and consensus-driven guidelines, standardized fluid management strategies are inconsistently applied across neonatal intensive care units (NICUs). A critical knowledge gap exists between evidence and practice. Early single-center studies suggest bundle feasibility and effectiveness but are limited in scope and generalizability. Incorporating implementation science frameworks and electronic health record (EHR) data pipeline integration can strengthen adoption, fidelity, adaptation, and sustainability of these interventions across diverse NICU settings. Pragmatic, multicenter studies that utilize EHR-based approaches are needed to help determine how to best implement functional fluid management strategies that improve patient-centered outcomes. Such bundles integrate evidence-based interventions that collectively identify high-risk patients, track, prevent, and treat FO. A structured pathway is needed to enhance scalability and uptake, systematically address barriers, tailor strategies to local contexts, and engage interdisciplinary teams. Bridging the gap between evidence and implementation through collaborative, pragmatic research has the potential to meaningfully reduce FO-related morbidity and mortality and advance neonatal critical care.

## 1 Introduction

**Figure 1:**
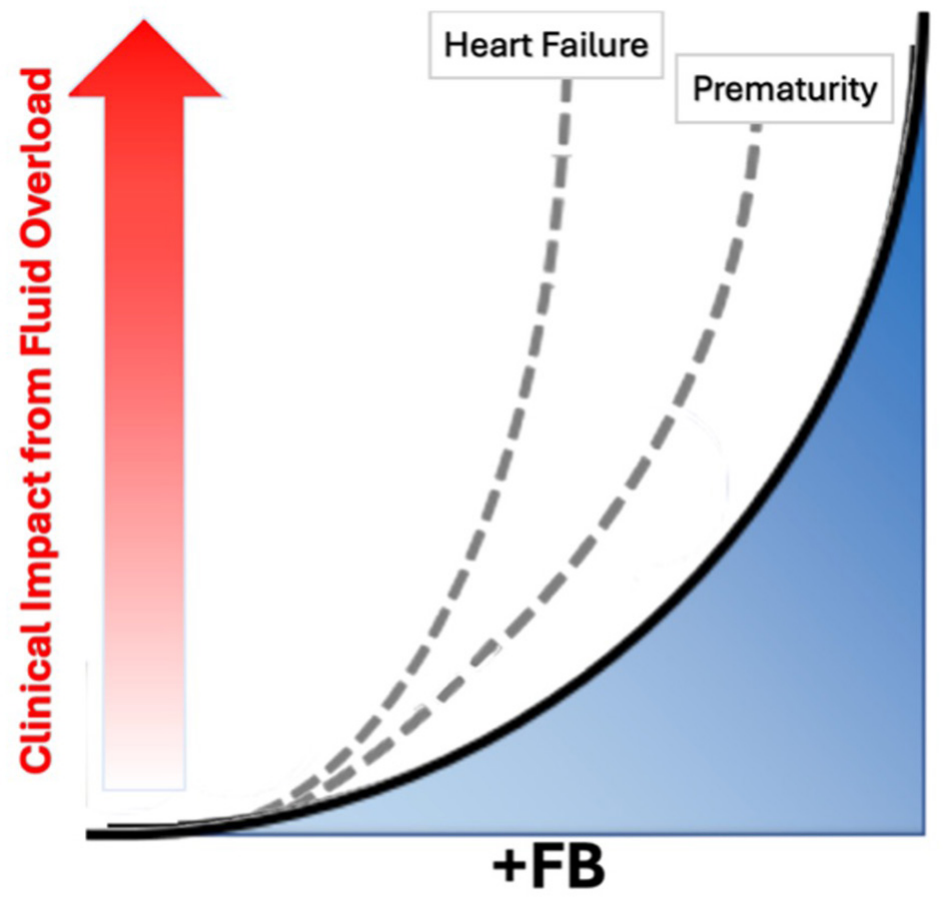
Relationship between fluid balance and overload and influence of patient factors.

Fluid balance (FB), the difference between fluid intake and output, often expressed as a percentage of the patient's weight (%FB), is a critical determinant of outcomes in critically ill patients. Adapted from the 2024 Acute Diseases Quality Initiative (ADQI) conceptual framework (1), Figure 1 highlights how patient factors, such as prematurity or heart failure, influence the relationship between cumulative FB (which may be positive or negative) and the clinical impact of fluid overload (FO). FO occurs when excess fluid causes congestion and impairs the function of multiple vital organs, including the lungs, heart, kidneys, and gastrointestinal system. Pulmonary fluid accumulation leads to interstitial and alveolar edema, impaired gas exchange, and the need for more intensive and prolonged mechanical ventilation (2–10). Paradoxically, prolonged ventilation can further damage the lungs through pressure injury, overdistension, inflammatory injury, and oxygen toxicity (11–21).

FO is a well-established, independent predictor of morbidity and mortality across neonatal, pediatric, and adult populations, as consistently shown in observational studies, systematic reviews, and meta-analyses (1–4, 6, 8, 10, 22–29). In neonates, the consequences are particularly profound, with strong evidence linking fluid accumulation to prolonged mechanical ventilation and increased mortality (2, 23, 25, 30–34). Despite growing understanding of FO, evidence-based interventions remain limited, especially in neonates, underscoring the need for pragmatic, multicenter studies that implement and evaluate standardized fluid management care bundles to improve outcomes in critically ill neonates.

In this perspective, we will outline the burden that FO has on clinical outcomes, review consensus guidelines and standard of care treatments, advocate for bundles of care and studies focused on them, discuss implementation science frameworks that should be utilized, and outline strategies for a bundle to best manage fluid overload. This article advocates for a shift toward broad pragmatic studies that integrate standardized fluid management care bundles within the EHR, leveraging implementation science frameworks to enable rigorous evaluation, fidelity monitoring, and advanced data analysis.

## 2 The burden and impact of fluid overload

### 2.1 Clinical consequences

The detrimental effects of FO, particularly on pulmonary function, are well documented across neonatal, pediatric, and adult critical care populations. Excess fluid leads to interstitial and alveolar edema, impairing gas exchange and leading to the requirement of more intensive and prolonged mechanical ventilation (6, 35–37). Beyond the lungs, FO increases cardiac preload and afterload, impairs renal perfusion, precipitates acute kidney injury, and contributes to gastrointestinal edema that can compromise nutrition. Observational studies in adults and children demonstrate associations between FO and increased ICU stays, higher vasoactive support requirements, sepsis-related complications, and mortality (12, 16–20).

Importantly, FO is not an inevitable consequence of critical illness, but a modifiable risk factor. Numerous studies have demonstrated fluid removal and proactive strategies, including careful monitoring of intake and output, judicious use of diuretics, and targeted fluid removal, to prevent FO could facilitate earlier liberation from mechanical ventilation, which can then decrease length of stay and improve patient outcomes (5, 22–24). These findings emphasize the critical importance of proactive strategies for early detection and effective, systematic management of FO in critically ill patients.

### 2.2 Evidence from neonatal populations

Over the last decade, scientific evidence of FO in neonates and its epidemiology has expanded (1, 2, 8, 10, 11, 13, 14, 24–26). Data from a 20+ center cohort of 874 premature neonates demonstrated that for each 10% increase in peak fluid balance, the odds of prolonged mechanical ventilation doubled (adjusted OR 2.03, 95% CI 1.64–2.51) (27). Recent systematic reviews and meta-analyses in neonates expand on these findings (2, 8, 11). One meta-analysis by Matushita et al. reported that FO increased mortality risk up to fivefold in neonates (13).

These studies consistently show that FO is a common complication in critically ill neonates and that FO contributes to adverse clinical outcomes. While the majority of data remains observational, the findings provide a strong basis for high-quality, targeted pragmatic interventions.

### 2.3 Importance to families, clinicians, hospitals, payors, and other stakeholders

**Figure 2:**
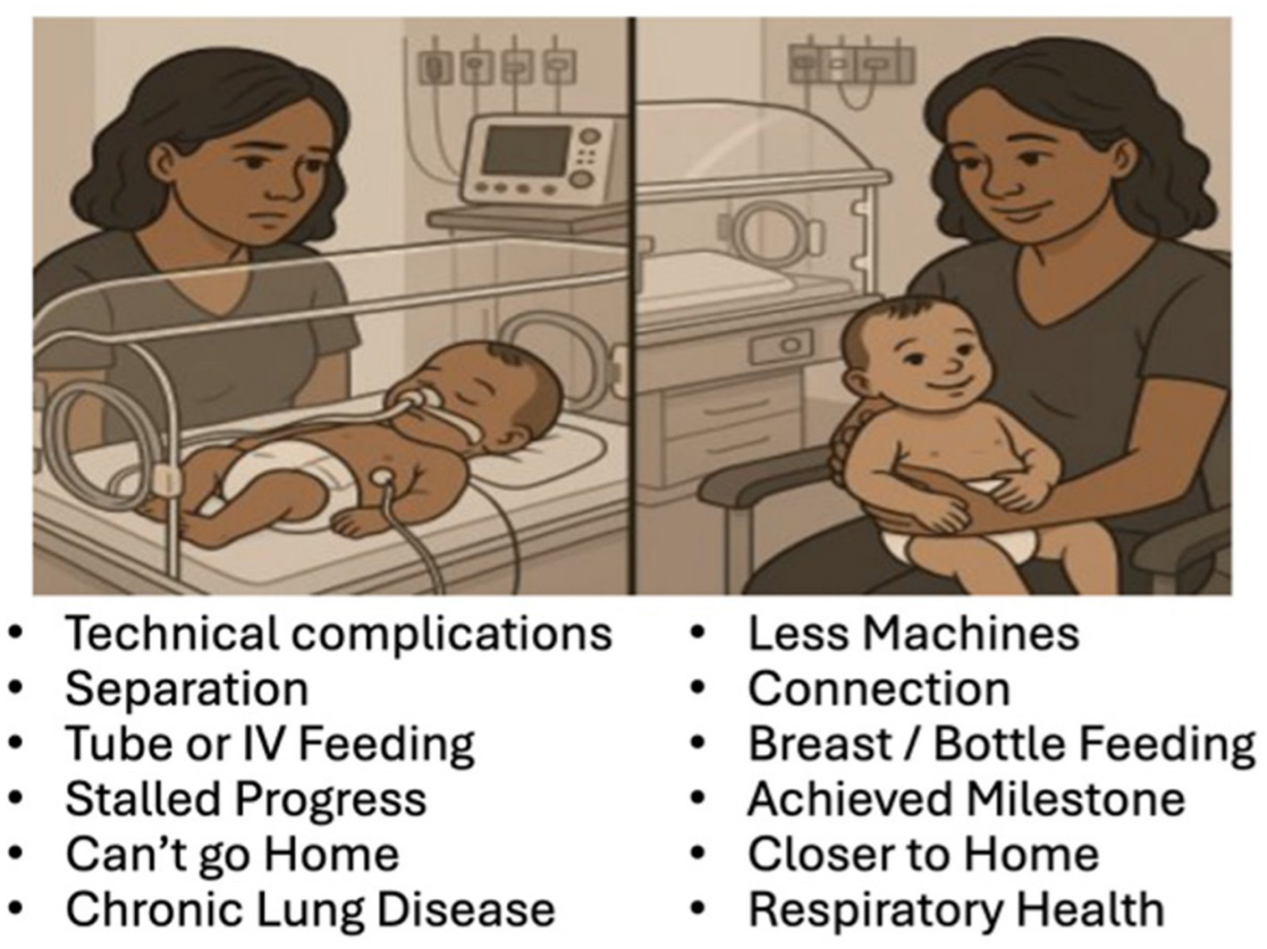
Reasons why families and others want to reduce ventilator days.

Beyond the direct clinical consequences, FO in critically ill neonates has profound consequences for families. Excess fluid contributes to pulmonary edema, prolonging the need for mechanical ventilation and increasing the risk of ventilation-related lung injury. Prior research shows that for parents and caregivers, the respiratory health of their infants is a primary concern, shaping both emotional wellbeing and perceptions of recovery progress (Figure 2). The Parent's Voice Project (38) and other family perspectives studies (39–42) consistently show respiratory health and earlier liberation from mechanical ventilation is important (11–20) not only because it reduces parental distress but also because a reduction in days on ventilator is associated with decreased emergency room visits, hospitalizations, and need for medications for chronic lung disease.

A reduction in the duration of mechanical ventilation is highly valued not only by families but also by clinicians, hospitals, payors, and other healthcare stakeholders. Prolonged mechanical ventilation is closely associated with an increased risk of chronic lung disease, including bronchopulmonary dysplasia, which can have long-term consequences for growth, development, and respiratory health. Extended ventilation also contributes to longer hospital stays, delaying discharge and increasing the exposure of vulnerable neonates to hospital-acquired complications (38–43).

From a systems perspective, prolonged mechanical ventilation drives substantial resource utilization, including increased nursing care, respiratory therapy, and monitoring requirements, while also placing demands on specialized equipment and intensive care capacity. These factors translate into higher healthcare costs, affecting hospitals' operational efficiency and financial sustainability, and influencing payor reimbursement and coverage considerations. Furthermore, prolonged ventilation may exacerbate disparities in care, as resource-intensive interventions are often more challenging to deliver consistently across diverse clinical settings (44–47). Therefore, strategies that safely reduce ventilator duration carry broad clinical, operational, and economic benefits, aligning with the priorities of families, healthcare teams, and the larger health system while supporting improved outcomes for critically ill neonates.

## 3 Evidence base for fluid management

### 3.1 Preventative and therapeutic therapies

A proactive approach to fluid management can reduce cumulative fluid balance and prevent the adverse clinical consequences of FO. Early identification and proactive fluid management, through careful monitoring, judicious diuretics, and targeted removal, improves oxygenation and shortens ventilation duration. Evidence from multiple studies demonstrates that decreasing cumulative FB improves oxygenation and shortens the duration of mechanical ventilation, directly impacting patient outcomes and supporting earlier liberation from respiratory support (5, 22–24).

### 3.2 Guidelines and consensus recommendations

Multiple critical care societies and expert groups emphasize the importance of prevention and treatment of FO in critically ill patients. Key guidelines and consensus statements include the Pediatric Surviving Sepsis Campaign (48), the pediatric Acute Diseases Quality Initiative (ADQI) (1), the Society of Critical Care Medicine's Fluid Management Group (49), recommendations for fluid management in ARDS (50), and the General Fluid Balance Language Consensus and others (48, 51–53). These efforts highlight both the clinical significance of FO and the urgent need for structured, evidence-based interventions across neonatal, pediatric, and adult critical care populations.

### 3.3 Practice variation in FO management highlights a gap between evidence and practice

Despite guidelines and recommendations, there has been poor adoption with wide variation in clinical practice, particularly in neonatal intensive care units (NICUs). A 2023 survey conducted by the Neonatal Kidney Collaborative (NKC) included 406 neonatal providers—primarily physicians (86%) and advanced practice providers (12%)—and revealed substantial heterogeneity in practice (54). The survey found that (54–57):

- No providers use a systematic approach to prevent and treat FO in their NICUs.
- Daily monitoring of intake/output and weights to calculate fluid balance is inconsistently performed.
- Few providers systematically initiate or titrate vasoactive medications to ensure adequate (58) urine output.
- Compared to pediatric and adult providers, neonatal clinicians typically tolerate higher degrees of positive FB before initiating interventions such as nephrology consultation or ultrafiltration (dialysis).

This variability underscores a critical gap between evidence-based recommendations and real-world practice, highlighting the need for standardized, scalable approaches to fluid management that can improve outcomes and reduce the burden of FO in critically ill neonates.

## 4 The case for bundled care approaches

There is a growing interest in bundled care approaches aimed at standardizing and improving fluid management practices in critically ill patients. Care bundles involve combining multiple standardized, evidence-based intervention strategies. In FO management, bundled care offers a way to integrate prevention, early detection, and treatment into an orchestrated intervention. Recent studies show that the implementation of bundles designed to systematically prevent and treat fluid overload can lead to meaningful improvements in outcomes. These study populations include critically ill cardiac patients, pediatric patients with shock who present to emergency departments, and children admitted to the PICU (58–65). In particular, Diaz et al. in 2018 (58) and Charaya et al. In 2025 (66) demonstrated that bundles specifically designed to reduce peak fluid balance in ventilated, critically-ill children lead to a reduction in length of stay and time on mechanical ventilation.

This evidence does not exclude neonates. In 2025, a unit-wide quality improvement initiative at the 48-bed Children's of Alabama Level IV NICU introduced structured approaches to prevent and mitigate FO (67). With consensus from all NICU providers, we implemented a bundle in infants who met criteria for a “high-risk of FO event”. These criteria included any one or more of: sepsis, new-onset hypotension, major surgeries, ≥ Stage 2 AKI, clinical edema, necrotizing enterocolitis, or spontaneous intestinal perforation. This study found:

- Improvement in Ventilator-Free Days (VFD) after bundle implementation (67 vs. 56% *p* < 0.001).
- Special cause variation with a 4% per month increase in VFD following introduction (*p* < 0.001).
- A reduction in median (IQR) length of stay after bundle [23 days (9–19, 19–49) vs. 19 (8–33) *p* < 0.02].
- No significant difference in demographics or balancing outcomes before and after bundle implementation.

This study shows that a systematic, proactive bundle designed to prevent and mitigate the clinical impact of FO is feasible, acceptable and effective (67).

### 4.1 Limitations and the need for multicenter pragmatic studies

This study, along with others like it, has important limitations. Many studies focused on this topic are single-center studies, were not conducted at level IV NICU's, and did not use rigorous study designs (i.e., randomized trials). Together, these factors limit both the internal validity (the confidence that observed effects are truly due to the intervention) and external validity (the applicability of findings across diverse clinical environments). As a result, important questions remain regarding the generalizability, reproducibility, and scalability of fluid management interventions in the NICU. Without multicenter, methodologically robust studies, it is challenging to determine whether observed benefits can be consistently achieved across NICUs of varying size, resources, and patient populations. These limitations underscore the urgent need for large-scale, pragmatic, and implementation-focused research to bridge the gap between promising single-site interventions and widespread improvements in neonatal care.

## 5 Improving study rigor with implementation science

### 5.1 Implementation science principles

To address these limitations and move toward scalable solutions, a deliberate implementation science framework should be adopted. Implementation science focuses on understanding the methods and strategies that facilitate the uptake of evidence-based interventions into routine practice. In the context of FO, this means validating the effectiveness of fluid management bundles while also ensuring they are adopted, implemented with fidelity, adapted appropriately, and sustained across diverse NICU environments.

### 5.2 Frameworks

Incorporating implementation science frameworks into the design and execution of bundled care studies strengthens both validity and real-world applicability. For instance, one foundational model, the Consolidated Framework for Implementation Research (CFIR), provides a pragmatic structure for understanding the complex, interacting factors that influence implementation in clinical settings (68). CFIR addresses domains such as intervention characteristics, organizational context, individual characteristics, and implementation processes. By systematically identifying and addressing barriers to adoption, tailoring strategies to diverse clinical environments, and evaluating outcomes across multiple dimensions, CFIR supports adaptation to local context, enhances scalability, and promotes sustainability. For example, a common barrier to the implementation of care bundles is the lack of workflow integration, which can be addressed by embedding the bundle as a clinical decision support tool into the electronic health record (EHR) (69). Prior research shows that implementation science frameworks are critical to identifying and addressing barriers to workflow integration, which results in improved uptake of care bundles and standardized evidence-based care (70). Ultimately, using an implementation science mindset and applying CFIR to fluid overload (FO) management ensures that bundled interventions are not only clinically effective but also feasible, scalable, and enduring across diverse NICU environments.

Complementary frameworks such as EPIS (Exploration, Preparation, Implementation, Sustainment) (71, 72) and RE-AIM (Reach, Effectiveness, Adoption, Implementation, Maintenance) (73, 74) add additional perspectives by structuring the phases of implementation and providing criteria to evaluate impact and sustainability. When used alongside frameworks like CFIR and EPIS, RE-AIM not only guides implementation planning but also systematically evaluates adoption, integration, and long-term effectiveness, ensuring that fluid management strategies translate into meaningful, sustained improvements in neonatal care.

### 5.3 Operationalizing Fluid Overload Bundles of Care through an EHR-Based Clinical Decision Support Tool

To translate a fluid management bundle into neonatal care, a functional data pipeline must be included to reliably capture the relevant clinical information, support timely treatment, and enable ongoing evaluation of both clinical effectiveness and implementation fidelity. Some of the key elements, and constructs that are needed include (75–78):

- Data capture: embedding bundle inputs into clinical decision support tools within the EHR minimizes the disruption to the clinical workflow and the risk of missed elements. Key data should be captured during normal rounds, allowing the tool to analyze reliable, clinical benchmarks and detect rising fluid balance.
- Clinical Decision Support (CDS): by interacting with the EHR to activate bundle elements when certain thresholds are met, providers are alerted of risk and able to make actionable treatment decisions swiftly.
- Execution of bundle: standardizing when and how clinicians evaluate fluid status reduces variation while allowing individualized responses. This combination directly addresses the key barrier of inconsistency in fluid management data and treatment that has existed in previous studies.
- Sustainability: using a CDS tool integrated with EHR's allows ongoing feedback, evaluation, and refinement to occur while monitoring implementation and fidelity to the bundle elements. On the back end, this structured data allows investigators and clinical teams to determine whether the bundle elements were delivered reliably. This also enables systematic evaluation of heterogeneity of treatment effect, allowing investigators to examine how the effectiveness of the bundle varies across diverse clinical settings, demographic groups, etc.

### 5.4 Defining the bundle

One of the most persistent challenges in developing fluid management bundles is determining which elements should be included without introducing unnecessary complexity. Implementation science, quality improvement theories, and pragmatic trials offer some strategies for constructing bundles that are both effective and implementable. Some of the most used methods for designing care bundles across a variety of topics are discussed below (76, 79–83):

- Outcome-Backward Design: this strategy selects bundle elements based on their relevance to outcomes that matter to patients, families, and health systems. In neonatal fluid overload, outcomes such as ventilator-free days, duration of mechanical ventilation, and length of stay provide clear clinical targets. Prioritizing these outcomes allows investigators to identify and prevent the failures most likely to exacerbate them. Each failure point becomes a bundle element (76, 79).
- Process Mapping to Identify Decision Points and Gaps: this strategy identifies where fluid management decisions occur and where gaps in care arise. By mapping how data such as intake/output, blood pressure, and urine output are collected, reviewed, and acted upon during daily care, teams can identify moments where FO is most likely to be overlooked or mismanaged. Decision points identified through process mapping present opportunities for targeted bundle prompts or clinical decision support. Aligning bundle elements within existing workflows improves compatibility and adoption (80).
- Iterative Refinement of Former Bundles: early bundle iterations often include enough elements to attempt to capture all potential contributors to fluid overload; however, evaluation often reveals that a certain few elements have the most clinical impact. Ongoing evaluation of bundle performance, fidelity, and impact allows the elimination of elements that are inconsistently followed, add burden, or do not influence clinical states. Removing or simplifying bundle elements enhances fidelity and sustainability without diminishing effectiveness (67, 80).
- Leveraging Implementation Frameworks to Enhance Uptake: combining trials with frameworks like CFIR, RE-AIM, and EPIS, allows for the assessment of adoption, implementation, and sustainability of bundles across diverse NICU settings. These approaches allow investigators to evaluate heterogeneity of treatment effect, identifying which patient subgroups benefit most from specific bundle elements and informing adaptations over time (81–83).

## 6 Discussion

The momentum behind FO research and clinical awareness is at a peak. The available observational data and single-site quality improvement reports have laid a solid foundation to target fluid management interventions in the ICU. However, to broadly bring meaningful improvements for critically ill patients at risk of FO, the critical care community needs multicenter broad pragmatic studies to show both the clinical effectiveness of integrated FO prevention strategies and the best implementation practices of these interventions.

Importantly, these studies should go beyond efficacy and explore implementation science principles, enabling investigators to understand how fluid management bundles can be best adopted, adjusted, and sustained across diverse NICU settings. Integrating bundles within the electronic health record (EHR) as clinical decision support tools is central to this effort. EHR integration enables standardized data capture, real-time decision support, and systematic monitoring of bundle delivery, while simultaneously generating high-resolution data to support robust evaluation.

EHR-enabled bundle implementation also facilitates rigorous analysis of heterogeneity of treatment effect, which allows researchers to move beyond average treatment effects and examine how bundle effectiveness varies across gestational ages, illness phenotypes, disease severity, and institutional contexts. By linking fidelity metrics, clinical actions, and outcomes within a unified data pipeline, investigators can identify which patients benefit most from specific bundle components and refine interventions accordingly. Understanding how treatment effects vary across subgroups of a population, researchers can determine more targeted and effective application of treatments, ultimately maximizing clinical impact while preserving scalability and sustainability.

## 7 Conclusion

Fluid overload in critically ill neonates is both common and consequential. To translate the decade of observational and consensus-driven evidence into improved clinical outcomes, the field needs a broad, pragmatic, multicenter study focused on implementing and evaluating a standardized fluid management care bundle in critically ill neonates. This approach has the potential to improve survival, reduce the burden of mechanical ventilation, and shorten the NICU stays for our most vulnerable patients.

## Data Availability

The original contributions presented in the study are included in the article/supplementary material, further inquiries can be directed to the corresponding author.
